# Evaluation of Internet-Based Crowdsourced Fundraising to Cover Health Care Costs in the United States

**DOI:** 10.1001/jamanetworkopen.2020.33157

**Published:** 2021-01-11

**Authors:** Suveen Angraal, Arun George Zachariah, Raaisa Raaisa, Rohan Khera, Praveen Rao, Harlan M. Krumholz, John A. Spertus

**Affiliations:** 1 Department of Internal Medicine, University of Missouri Kansas City School of Medicine, Kansas City; 2 Department of Electrical Engineering and Computer Science, University of Missouri, Columbia; 3 Department of Internal Medicine, Yale School of Medicine, New Haven, Connecticut; 4 Section of Cardiovascular Medicine, Department of Internal Medicine, Yale School of Medicine, New Haven, Connecticut; 5 Center for Outcomes Research and Evaluation, Yale-New Haven Health, New Haven, Connecticut; 6 Department of Health Management and Informatics, University of Missouri, Columbia; 7 Department of Health Policy and Management, Yale School of Public Health, New Haven, Connecticut; 8 Health Outcomes Research, St Luke’s Mid America Heart Institute, University of Missouri-Kansas City, Kansas City

## Abstract

This serial cross-sectional study examines online crowdsourced fundraising for US health care costs from 2010 to 2018 and evaluates whether there are trends by medical condition and geographic distribution.

## Introduction

Online fundraising platforms have emerged as means to raise money for charity. Patients can also access these platforms to receive charitable contributions to support their health care costs. We sought to evaluate the use of a popular fundraising platform to cover health care–related costs, the medical conditions involved with these fundraisers, and their geographic distribution in the US.

## Methods

This serial cross-sectional study was exempted from review by the University of Missouri Kansas City institutional review board because it did not contain patient data. Participant consent was waived because all data were publicly available.

We extracted data from the GoFundMe website, from its inception in May 2010 through December 2018. A looping web scraper tool was created^1^ to extract the following data: text body of the fundraiser, self-tagged category, geotagged location, date of creation, target amount sought (in US dollars), and total amount raised (in US dollars). We ran the program in April 2019. Fundraisers self-tagged as medical were included.

We classified fundraisers by key conditions that pose high morbidity in the US: cancer, cardiovascular conditions, neurological conditions, and trauma or injury.^2^ For classification, clinical descriptors from the main text body of these fundraising campaigns were extracted using a pretrained machine learning model,^3^ and campaigns were then categorized into disease categories using a natural language processing algorithm through biomedical word vectors.^4^ A manual abstraction of 1000 randomly selected fundraisers found a 90.1% classification accuracy. Geographic tags were used to assess state-level distribution, standardized to 2010 US Census data. Finally, using linear regression, we compared the state-level prevalence of online fundraisers with the Charitable Giving Index to assess how charity patterns correlate with online fundraisers.^5^ Two-tailed *P* < .05 was considered statistically significant. Data analyses were performed using Python software version 3.6 (Python Software Foundation) and R statistical software version 3.6.0 (R Project for Statistical Computing) from July 2019 to February 2020 (eMethods in the Supplement).

## Results

**Figure 1.**
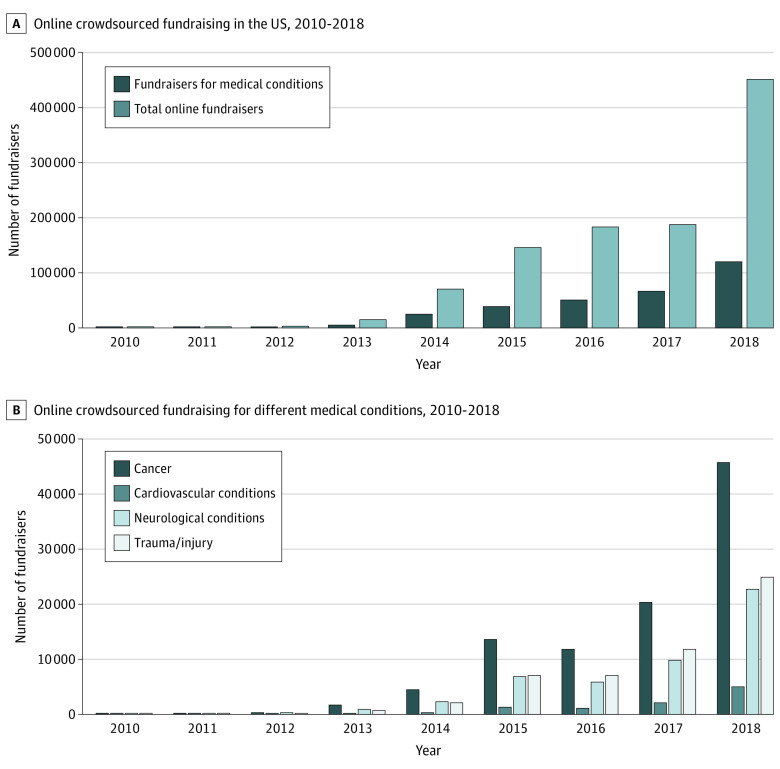
Online Crowdsourced Fundraising Trends for Medical Conditions in the US, 2010-2018

Of the 1 056 455 fundraisers on the online platform in the US between May 2010 and December 2018, 281 881 (26.7%) were created to cover health care–related costs, collectively seeking $10 285 738 233. As of April 2019, $3 663 935 620 had been raised. There was a large increase in the use of medical fundraisers over time; from 42 fundraisers in 2010 to 119 373 in 2018 (a mean increase of 14 916 fundraisers per year) (Figure 1A). In 2010, $717 125 was sought, which increased to $4 663 513 572 in 2018 (mean increase of $582 849 556 per year).

A total of 98 352 fundraisers (34.9%) were for cancer, 53 861 (19.1%) for trauma/injury, 48 963 (17.4%) for neurological conditions, and 10 143 (3.6%) for cardiovascular conditions. The number of online medical fundraisers increased for all 4 conditions over the study period (Figure 1B). For cancer, $4 481 980 170 was sought ($45 571 per fundraiser). For trauma/injury, $1 609 046 833 was sought ($29 874 per fundraiser). Neurological and cardiovascular conditions sought a total of $1 212 452 440 and $287 113 426 ($24 763 and $28 307 per fundraiser, respectively).

**Figure 2.**
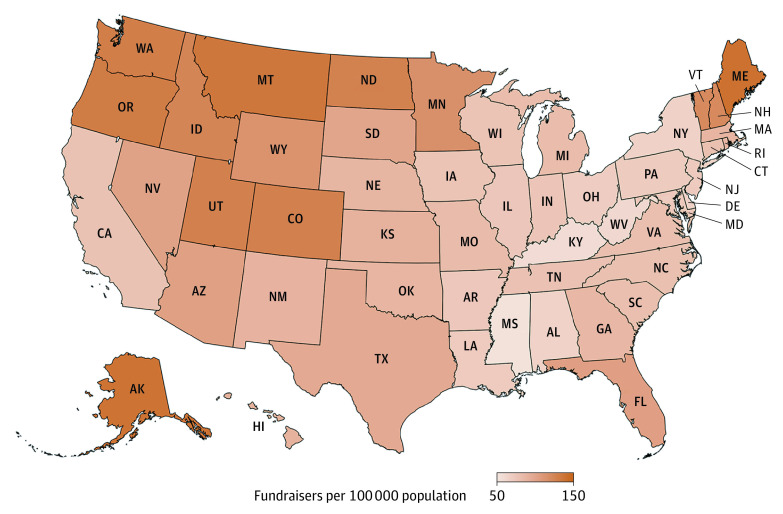
Geographical Distribution of the Use of Online Crowdsourced Fundraising for Medical Conditions in the United States

Maine had the highest prevalence of online medical fundraisers (139.4 fundraisers per 100 000 population), followed by Alaska (137.2 fundraisers per 100 000 population) (Figure 2). Mississippi had the lowest prevalence of online medical fundraisers (54.6 fundraisers per 100 000 population). The states with higher Charitable Giving Index had a higher prevalence of online fundraisers (*β* = 0.072; *P* = .03; *β*=0.072).

## Discussion

From May 2010 through December 2018, more than $10 billion was sought through online medical fundraisers in the US, with more than $3 billion raised. Cancer represented the most common medical condition for which funding was sought, followed by trauma/injury.

Cancer therapy is expensive, and out-of-pocket costs for newly diagnosed patients with cancer frequently represent 23% to 63% of their household income.^6^ Our study suggests that many patients are using online fundraisers to cope with the high financial burden due to cancer.

This study had some limitations. Although our study does not contain patient-specific clinical data and included only 1 fundraising platform, thereby representing the lower bound on the true use of such mechanisms, it highlights a unique aspect of financial toxicity of health care.

Online fundraising to cover health care–related expenditures has grown substantially over the past years. These results highlight how many people are relying on the charity of others for raising money to cover health care costs.

## Notes

Supplement. **eMethods.** Additional Methodology

## References

[1] beautifulsoup4 4.8.2. The Python Package Index. Published 2019 Accessed November 9, 2020. https://pypi.org/project/beautifulsoup4/

[2] Mokdad AH, Ballestros K, Echko M, ; US Burden of Disease Collaborators The state of US health, 1990-2016: burden of diseases, injuries, and risk factors among US states. JAMA. 2018;319(14):1444-1472. doi:10.1001/jama.2018.0158 29634829 PMC5933332

[3] Zhu H, Paschalidis IC, Tahmasebi A Clinical concept extraction with contextual word embedding. arXiv. Preprint posted 2018 Accessed November 9, 2020. https://arxiv.org/abs/1810.10566

[4] Zhang Y, Chen Q, Yang Z, Lin H, Lu Z BioWordVec, improving biomedical word embeddings with subword information and MeSH. Sci Data. 2019;6(1):52. doi:10.1038/s41597-019-0055-0 31076572 PMC6510737

[5] McCann F Most charitable states. WalletHub Published 2019 Accessed November 9, 2020. https://wallethub.com/edu/most-and-least-charitable-states/8555

[6] Ekwueme DU, Zhao J, Rim SH, Annual out-of-pocket expenditures and financial hardship among cancer survivors aged 18-64 years—United States, 2011-2016. MMWR Morb Mortal Wkly Rep. 2019;68(22):494-499. doi:10.15585/mmwr.mm6822a2 31170127 PMC6553808

