# Supplementary material for: Evaluation of Internet-Based Crowdsourced Fundraising to Cover Health Care Costs in the United States
Source: JAMA Netw Open. 2021 Jan 11;4(1):e2033157. doi: 10.1001/jamanetworkopen.2020.33157 (PMC7801937; doi:10.1001/jamanetworkopen.2020.33157)
Supplement: Supplement. — eMethods. Additional Methodology [file jamanetwopen-e2033157-s001.pdf]

Supplemental Online Content

Angraal S, Zachariah AG, Raaisa R, et al. Evaluation of internet-based crowdsourced fundraising to cover health care costs in the United States. *JAMA Netw Open*. 2021;4(1):e2033157. doi:10.1001/jamanetworkopen.2020.33157

eMethods. Additional Methodology

This supplemental material has been provided by the authors to give readers additional information about their work.

© 2021 Angraal S et al. *JAMA Network Open.*

eMethods. Additional Methodology

Evaluation of Internet-based Crowdsourced Fundraising to Cover Healthcare Costs in the United States

Angraal, et al.

Data extraction

Data from the largest online fundraising platform (GoFundMe) was extracted. For this, we designed a custom looping web scraper tool that extracted data elements from all fundraisers. This web scrapper was developed using the *BeautifulSoup* Python package,[1] which allows for automated navigating, searching, and modifying a parse-tree for downloading data from a webpage. Data were extracted from both active and inactive fundraisers. Extracted elements included the main text body of the fundraiser, geotagged location, date of creation of the fundraiser, target amount sought ($ US), and the total amount donated ($ US). A random sample of 1000 URLs was selected for pilot assessment of the web scraper algorithm. Data were then collected from these 1000 URLs. A prespecified schema was used to assess the accuracy of the data capture through the scraper algorithm. The web scraper captured 100% of the data for the aforementioned variables. We ran the program in April 2019. Fundraisers that were self-tagged as 'Medical' were included in the analysis. The code has been made available for public access on GitHub (https://github.com/lmeninato/GoFundMe).

Descriptive analysis

We used the creation dates of fundraisers to assess the annual trends in the use of online crowdsourcing. We aggregated the amount of funding sought, and the amount donated for these medical fundraisers to assess annual funding trends. No inflation adjustments were applied, given the relatively short duration of observation and the low rates of inflation in the US during that time. Further, we identified the medical fundraisers by key conditions that pose high morbidity in the United States: cancer, cardiovascular diseases, neurological diseases, and trauma or injury.[2]

Classification of the fundraisers

© 2021 Angraal S et al. *JAMA Network Open.*

For classification, we used a 2-step approach. First, clinical entities from the main text body of these fundraisers were extracted using a machine learning model we developed.[3] This model was trained on clinical reports and Wikipedia data to account for common medical language. The model was validated on data from the 'Integrative Biology and the Bedside' platform for clinical accuracy. This model extracts the clinical entities from the main text body of the fundraisers through contextual word embedding. Secondly, we developed a natural language processing (NLP) algorithm to classify these extracted clinical entities in the following categories: cancer, cardiovascular diseases, neurological diseases, trauma/injury, or other conditions. This NLP classification model was trained on data from PubMed and Medical Subject Headings thesaurus, a hierarchical medical vocabulary produced by the National Library of Medicine.[4] Using this model, the extracted clinical entities from the first model were mapped using word vectors for accurate classification in the aforementioned disease categories. F1 score and Spearman's rank coefficients were used to assess the accuracy of the machine learning model and the NLP algorithm, respectively. The machine learning model showed an F1 score of 88.6 over the 2010 i2b2/VA challenge dataset curated for three different tasks, which involves extracting medical concepts from patient records, classifying medical problems and identifying the relations between problems, tests and treatments. The NLP algorithm trained over the PubMed and MeSH corpus, showed a Spearman's rank correlation coefficient score of 0.657 and 0.617 over the UMNSRS Similarity and Relatedness datasets, respectively. A manual abstraction of a random sample of 1000 fundraisers revealed that the classification algorithm had 90.1% accuracy.

Geographical variation

Geographic tags were used to identify the states of origin of the fundraisers created in the United States. We normalized the number of fundraisers to the population of each state. State-level geographical distribution of the use of online medical fundraisers was measured in the number of online medical fundraisers per 100,000 population using 2010 United States Census data.[5] States with most and least fundraisers per 100,000 population were identified.

This study was exempt from the institutional review board approval. Python version 3.6 and R programming language version 3.6.0 was used for the analysis. Patients and the public were not involved in the production of this research.

© 2021 Angraal S et al. *JAMA Network Open.*
